# Bright photoluminescence from ordered arrays of SiGe nanowires grown on Si(111)

**DOI:** 10.3762/bjnano.5.259

**Published:** 2014-12-30

**Authors:** D J Lockwood, N L Rowell, A Benkouider, A Ronda, L Favre, I Berbezier

**Affiliations:** 1 Measurement Science and Standards, National Research Council, 1200 Montreal Road, Ottawa, Ontario K1A 0R6, Canada; 2 CNRS, Institut Matériaux Microélectronique Nanosciences de Provence, AMU, Avenue Normandie Niemen, 13397 Marseille Cedex 20, France

**Keywords:** bandgap, germanium, nanowires, near field, silicon, photoluminescence

## Abstract

We report on the optical properties of SiGe nanowires (NWs) grown by molecular beam epitaxy (MBE) in ordered arrays on SiO_2_/Si(111) substrates. The production method employs Au catalysts with self-limited sizes deposited in SiO_2_-free sites opened-up in the substrate by focused ion beam patterning for the preferential nucleation and growth of these well-organized NWs. The NWs thus produced have a diameter of 200 nm, a length of 200 nm, and a Ge concentration *x* = 0.15. Their photoluminescence (PL) spectra were measured at low temperatures (from 6 to 25 K) with excitation at 405 and 458 nm. There are four major features in the energy range of interest (980–1120 meV) at energies of 1040.7, 1082.8, 1092.5, and 1098.5 meV, which are assigned to the NW-transverse optic (TO) Si–Si mode, NW-transverse acoustic (TA), Si–substrate–TO and NW-no-phonon (NP) lines, respectively. From these results the NW TA and TO phonon energies are found to be 15.7 and 57.8 meV, respectively, which agree very well with the values expected for bulk Si_1−_*_x_*Ge*_x_* with *x* = 0.15, while the measured NW NP energy of 1099 meV would indicate a bulk-like Ge concentration of *x* = 0.14. Both of these concentrations values, as determined from PL, are in agreement with the target value. The NWs are too large in diameter for a quantum confinement induced energy shift in the band gap. Nevertheless, NW PL is readily observed, indicating that efficient carrier recombination is occurring within the NWs.

## Introduction

Semiconductor nanowires (NWs) are thought of as promising building blocks for opto-electronic devices that exploit their novel electronic band structures generated by two-dimensional (2D) quantum confinement in conjunction with their associated optical properties [1–6]. However, in order to fully implement these new properties, strict control is needed over the NW location, uniformity, composition, and size. By exploiting such band gap engineering, directly allowed transitions have been demonstrated for specific core/shell NW configurations with an ultimate control over the NW shape, aspect ratio and radial multishell composition [7]. A major asset of Si/Ge core/shell [8] and axial [9] NW heterostructures is also their ease of integration in CMOS technology, which allows the fabrication of opto-electronic devices with an increased photon absorption over a wider range of wavelengths and with an improved efficiency of electron generation. In addition, by combining the extraordinary technological know-how that has been developed for Si with direct-gap Si/Ge heterostructures, the fabrication of various NWs for applications such as photovoltaic tandem solar cells has been enabled [10–12]. Most of the device specifications require a low cost fabrication process with good control over the NW reproducibility and uniformity [13].

A variety of different NW growth methods have been reported including vapor–liquid–solid [14–17], solid–liquid–solid [18–19], vapor–solid–solid [20–22], oxide-assisted [23], and others [24–27]. Many of these growth methods have lead to NWs possessing non-uniform diameters and lengths and that are haphazardly oriented and randomly positioned [28]. We have evolved an efficient and simple electrochemical process that joins focused-ion-beam (FIB) lithography and galvanic reaction to selectively prepare gold nanoparticles in well-defined locations. Afterwards these nanoparticles are used for the molecular beam epitaxy (MBE) growth of ordered SiGe NW arrays with predefined NW positions and diameters. Here we report on the optical properties of such MBE grown NWs.

## Experimental

**Figure 1:**
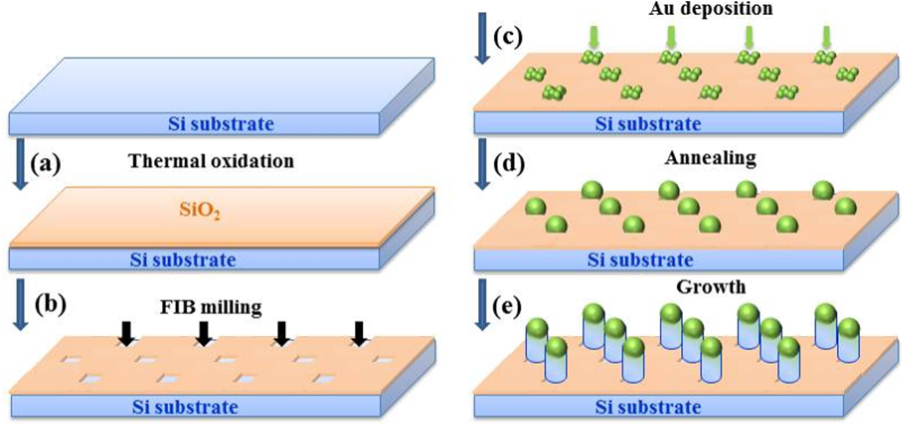
Schematic representation of the process steps: (a) formation of SiO_2_ (5 nm thick) by RTO; (b) opening of SiO_2_-free windows by FIB milling; (c) Au deposition by oxido-reduction of gold salts; (d) phase transition of Au in AuSi clusters by annealing at temperature *T*_A_; and (e) MBE growth of SiGe NWs at *T*_A_.

A schematic overview of the various steps used in the growth process is given in Figure 1. The steps consisted of: (a) rapid thermal oxidation (RTO); (b) FIB patterning; (c) galvanic selective deposition of Au; (d) induced phase transition in AuSi catalysts; and (e) selective growth of SiGe NWs. With this method we have produced Si_1−_*_x_*Ge*_x_* NWs with diameters in the range 50–200 nm, although the size can potentially be tuned between 30 and 300 nm, and with Ge concentration *x* in the range 0 ≤ *x* ≤ 0.15.

Prior to the substrate patterning (Figure 1a), the Si(111) substrates, which were either 5 cm diameter wafers or wafer sections of dimensions 2 × 2 cm^2^ and 1 × 1 cm^2^, were first systematically cleaned by a modified Shiraki ex situ process and then capped with an ultra-thin (5 nm thick) SiO_2_ thermal oxide (UTO) that was obtained by rapid thermal oxidation (RTO) in a clean vacuum.

In the second step (Figure 1b), 2D arrays of small windows (with diameters in the range 50–200 nm) were opened in the UTO by FIB milling using a Tescan LYRA1 XMH dual-beam FIB workstation having an Orsay Physics mass filtered ion column operated at 30 keV. A liquid metal alloy ion source (LMAIS) of Au_4_Si ([Si] = 19%, [Au] = 81%) heated at 450 °C was used for the milling step; a Au^2+^ or Si^+^ ion beam was selected independently by a Wien filter. The patterns in the Si/SiO_2_ substrate were milled with the Au^2+^ ion beam at an incident angle of ≈10° from the normal: Regarding the choice of incident angle, we have shown in another study [29] that the sputtering rate is larger when working at 10° from the normal. The FIB milling process should be performed with a low current dose to minimize the surface roughening of the substrate and the density of defects formed; typically, the emission current used was about 10 pA and the ion dose = 10^16^ PA/cm^2^/s. This point is essential to provide an efficient selectivity during the last NW growth step.

After the FIB etching, the samples were immediately dipped into a gold salt (H^+^Au^3+^Cl_4_^−^) aqueous solution at room temperature. Since the freshly FIB etched nanopits on silicon are then SiH_x_^−^ terminated, while the original surface remains coated with the inert thermal SiO_2_ layer, the interiors of the nanopits are ready for the electrochemical step. The latter step, called galvanic deposition, is based on the spontaneous oxido-reduction reactions between the semiconductor surface (the substrate) and the metallic ions in the solution. Upon contact with the Si surface, the solution spontaneously reduces and precipitates into Au nanocrystals, according to the following equation:

[1]
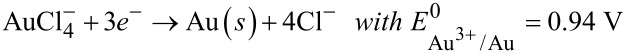


In parallel, in the aqueous solution, as a result of the high reduction potential of gold ions, the Si surface, which provides the electrons to reduce the gold ions to metallic gold, oxidizes into SiO_2_ as per the following equation:

[2]
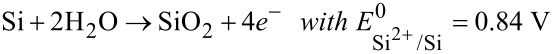


This spontaneously formed silicon dioxide prevents further metal ion reduction. Since the gold salt reduction process is not possible on SiO_2_, the reaction immediately stops after the formation of SiO_2_ [30].

After the selective galvanic deposition of gold clusters on the substrate, the samples were loaded into the MBE chamber. The phase transformation from the small Au nanoclusters located within the SiO_2_-cover-free nanopits to the Au_0.18_Si_0.82_ eutectic alloy is obtained by thermal annealing at 550 °C for 30 min.

**Figure 2:**
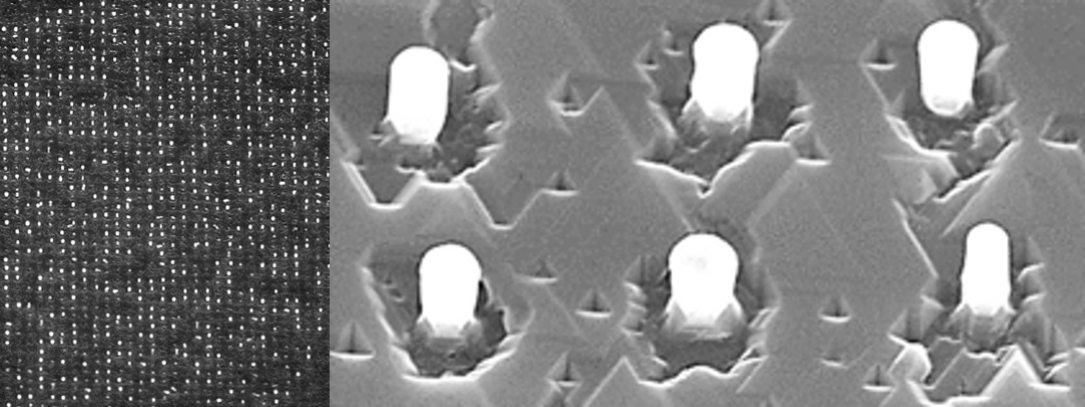
Scanning electron microscope (SEM) images of the ordered arrays of Si NWs showing (left) a NW array and (right) individual 200 nm long NWs.

The annealing and growth experiments were performed in the MBE growth chamber of a Riber SIVA32 system with a base pressure of 10^−11^ Torr. The silicon flux was obtained from an electron beam evaporator and maintained constant during the deposition at 0.03 nm/s, while germanium was deposited from an effusion cell. The growth temperatures were varied between 380 and 600 °C and were controlled in real time using an infrared pyrometer. The silicon substrate was rotated during the experiments to maintain a thickness and composition uniformity over the whole wafer or wafer section (the results obtained were similar for all substrates). Figure 2 displays SEM images of representative Si NWs grown at 550 °C on the positionally-ordered Au_0.18_Si_0.82_ catalysts. The main advantage of this growth method is the control of the NW position (related to site selectivity) and its size; a homogeneous size is obtained due to the regular network of Au nanocrystals (see Figure 2). Also, SiGe NWs can be grown and then transformed in a second step into core-shell NWs using a condensation process that we have developed.

Three NW samples were prepared for this study: Sample (A), where the NWs are grown randomly across the Si substrate; sample (B), where the nanowires decorate the edges of 400 × 400 µm^2^ boxes; and sample (C), where the NWs fill 400 × 400 µm^2^ boxes in ordered arrays, as described above. These samples have NWs that have a nominal Ge concentration of *x* = 0.15 and that are 200 nm in diameter and 200 nm long, with a morphology similar to the Si NWs shown in Figure 2.

The photoluminescence (PL) spectra were measured at low temperatures (from 6 to 25 K) using a Bomem DA3 FTIR spectrometer equipped with a cooled Ge (Applied Detector Corporation) detector, and the samples were excited with loosely-focused light from a GaN-based semiconductor laser (70 mW at 405 nm) or from an argon ion laser (35 mW at 458 nm).

## Results and Discussion

**Figure 3:**
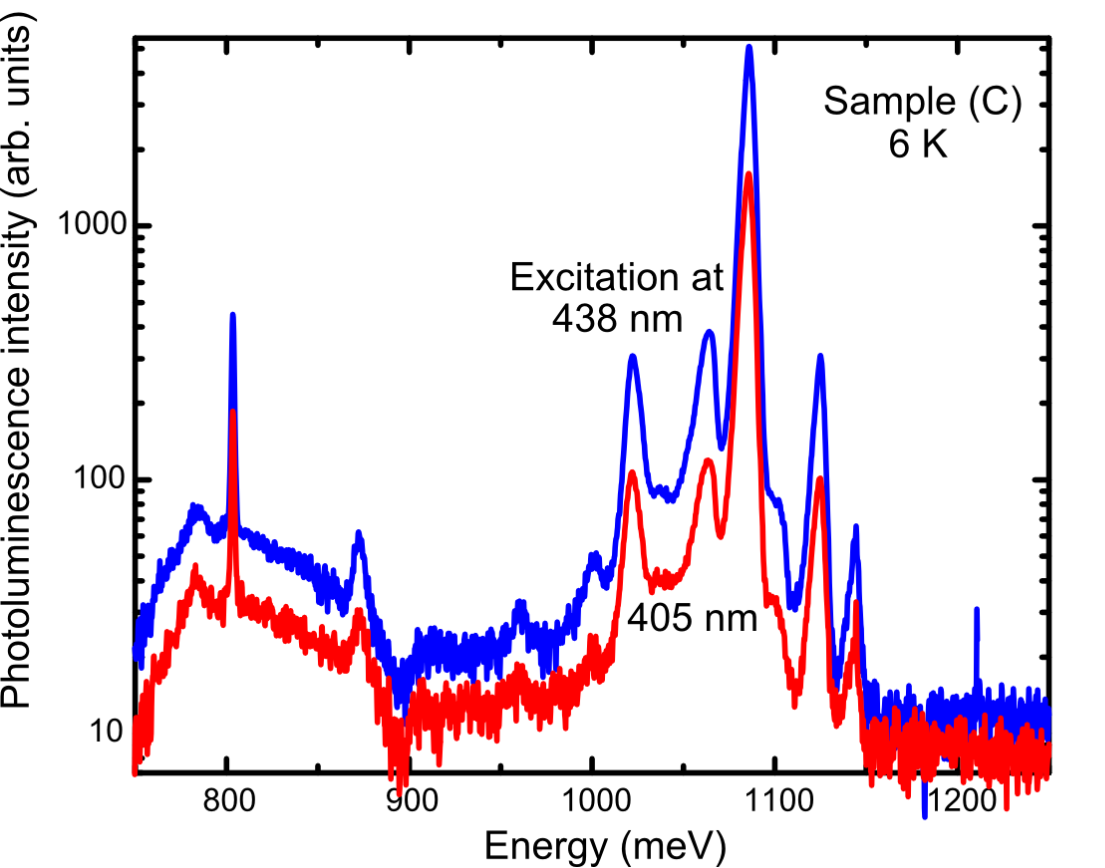
The raw PL spectrum obtained from sample (C) at 6 K with excitation at 405 and 458 nm.

The PL spectra obtained for sample (C) with excitation at 405 and 438 nm are compared in Figure 3. The two spectra exhibit the same features, most of which arise from the Si substrate together with a few arising from instrumental effects. The main difference between them, as also observed for the other two samples at 6 K, lies in their overall intensity: the spectrum excited at 458 nm is much more intense than the 405 nm one. This is a consequence of the exciting light at 438 nm penetrating beyond the NWs further into the substrate than the 405 nm light and thus enhancing the Si PL intensity. The NW features we are interested in overlap the Si PL, and thus to help minimize the substrate signal we consider next only results obtained using excitation at 405 nm.

**Figure 4:**
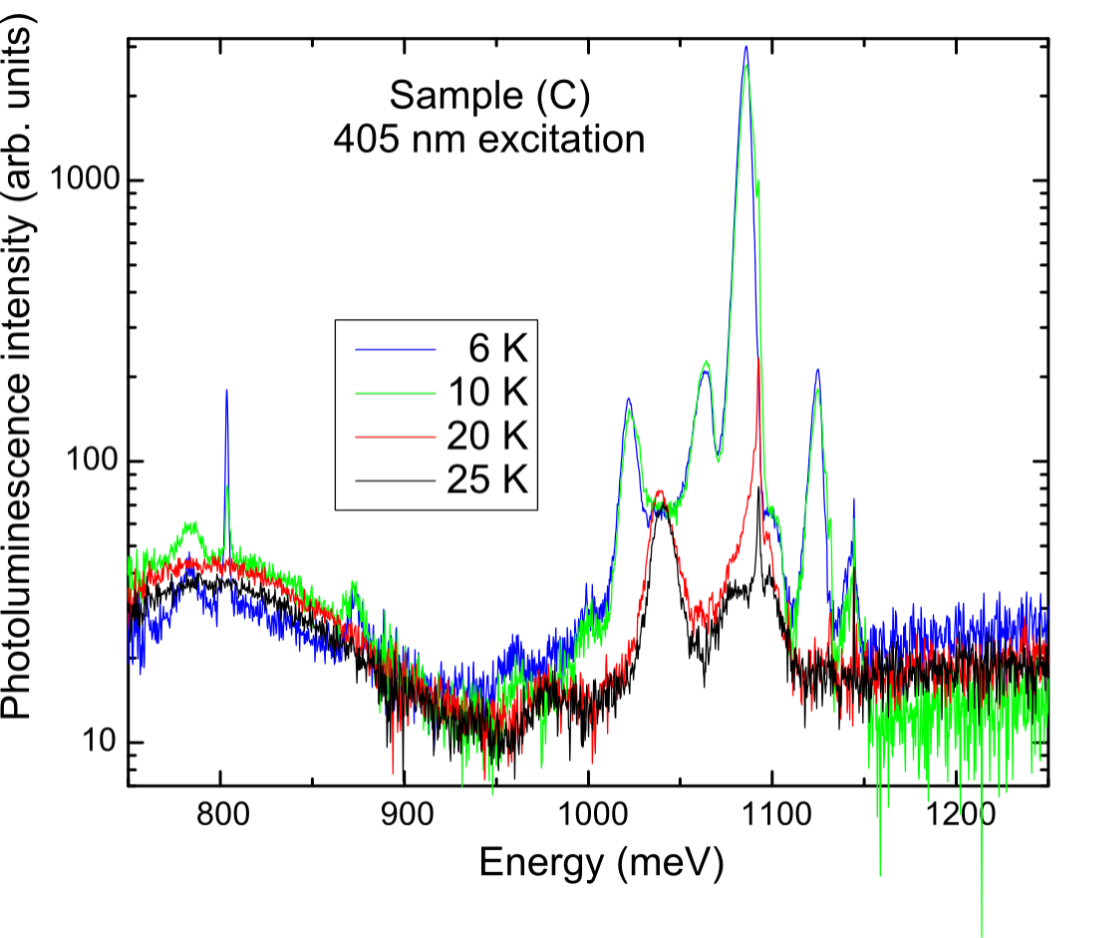
Temperature dependence of the instrument-response-corrected PL spectrum obtained from sample (C) with excitation at 405 nm.

The temperature dependence of the PL spectrum obtained from sample (C) is shown in Figure 4. PL spectra with similar temperature dependences were obtained from the other samples. Figure 4 shows that the NW spectral region of interest (from approximately 950 to 1050 meV) is dominated by the boron (≈10^17^ cm^−3^)-doped Si substrate phonon-replica spectrum at the lowest temperatures (6 and 10 K). On increasing the sample temperature up to 20 K, the Si substrate PL becomes sufficiently quenched from the increasing dissociation of multiple-donor bound excitons within the substrate [31] that the underlying NW PL is more readily seen. By 25 K, only the sharp line at 1092.5 meV due to the Si substrate remains. From now on we shall consider only the PL obtained from the samples at 25 K to avoid the overlapping substrate signal as much as possible.

**Figure 5:**
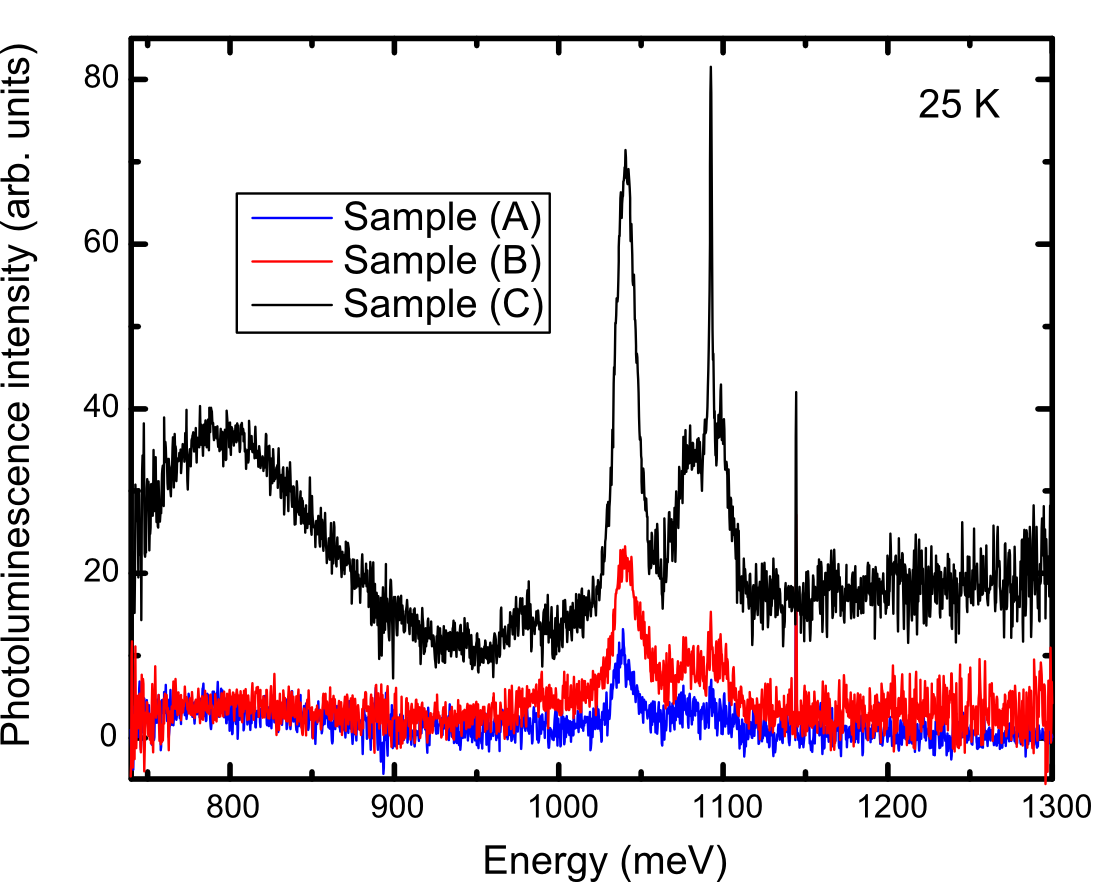
Instrument-response-corrected PL spectra obtained with 405 nm excitation from the (A), (B), and (C) samples at 25 K.

The instrument-response-corrected PL spectra obtained from all three samples at 25 K with excitation at 405 nm are given in Figure 5. At energies below the range of interest for the NWs, broad features are seen at ≈800, 940, and 975 meV that are due to the Si substrate. At higher energies, there is a very sharp peak at 1144 meV associated with an instrumental emission line that should be ignored. The overall intensity of the NW PL varied from sample to sample, as can be seen in Figure 5; sample (C), with a higher density of NWs distributed within the array, was the strongest, while sample (A) with a random distribution of NWs was the weakest. Nevertheless, the spectra are quite similar overall.

The NW PL is strong considering that the volume of NW material is so small (each wire has a volume of 0.006 µm^3^) and thus the carriers have to be recombining efficiently within the wires to produce this light emission at the SiGe-alloy band gap energy. We know from earlier studies of SiGe etched wires [32] and dots [33] that just the spatial confinement of carriers is sufficient to produce the readily-observed PL found here, which implies that the carriers in these samples are not being lost in large numbers to the substrate or recombining in large quantities at defects inside or on the surface of the NWs. The wires are too large in diameter for a quantum confinement induced energy shift in the band gap, but the phonon energies could be affected slightly by confinement and surface effects [34]. The wires are grown free-standing and thus there should be no internal strain (i.e., bulk-like energy values should be observed).

**Figure 6:**
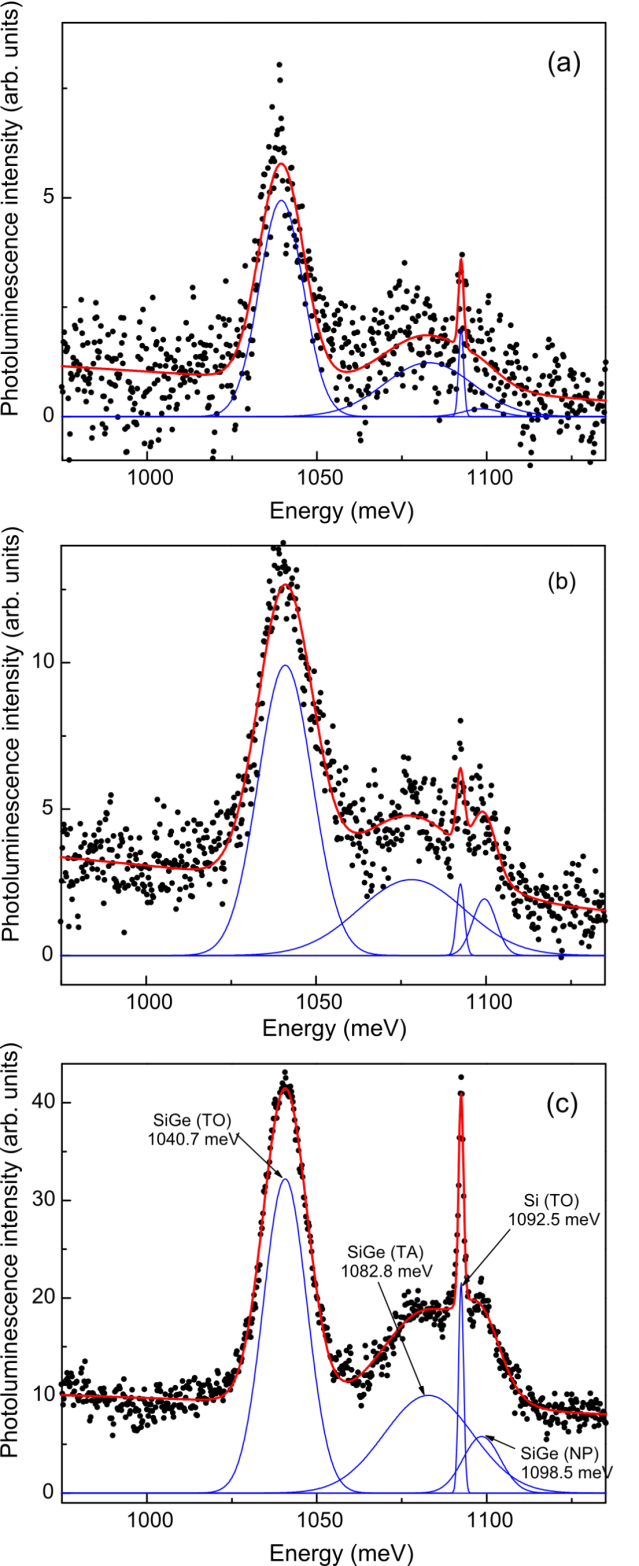
Curve-resolved PL spectrum of (a) sample (A), (b) sample (B), and (c) sample (C) at 25 K obtained with 405 nm excitation. The solid line shows the overall fit to the PL data, while the three SiGe NW component lines are shown beneath the fitted spectrum. The very sharp line at 1092.5 meV arises from the Si substrate.

Further details about the PL spectra can be obtained from spectral curve resolving, Such an analysis using a Gaussian line shape revealed that there are four major features in the energy range of interest (see Figure 6 for results obtained for the three samples at 25 K). The fitted peak energies, line widths, and amplitudes for the three samples are given in Table 1. In order of increasing energy, they are readily assigned to the NW free-exciton transverse-optic (TO) Si–Si vibrational mode, NW free-exciton transverse-acoustic (TA) phonon, Si–substrate-bound-exciton TO phonon and NW free-exciton no-phonon (NP) lines, respectively [31,35]. The peak amplitude data given in Table 1 can be used to estimate the amplitude ratios of the NW lines relative to the strongest line (the NW TO mode) for each sample and the results are given in Table 2. Given the uncertainties in the fits obtained from curve resolving, the amplitude ratio for the TO/TA peaks is much the same in the three samples (see Table 2), as would be expected if both lines arose just from the NWs and that the NWs were of similar composition in all samples. The good agreement obtained between the TO/TA peak amplitude ratios for Samples (B) and (C) is not as good for Sample (A), whose PL was much weaker than the other two samples resulting in greater errors in the peak amplitudes from the fits. The fitted frequencies and line widths for the respective NW and Si TO mode lines vary slightly between the three samples, but are essentially the same within error (see Table 1). Interestingly, the free-exciton NP line intensity relative to its phonon replicas (TA and TO lines) in these NWs is much more intense than that found in bulk Si, but is somewhat lower compared to what is observed in bulk alloy material of a similar composition [35], which may reflect on the action of NW surface effects as opposed to the usual alloy disorder effect resulting in a breakdown in the wave vector selection rules.

**Table 1:** Results of curve resolving the PL spectra excited with 405 nm excitation for the three NW samples at 25 K. The fitted band frequencies ω_i_ and widths γ_i_ are given in millielectronvolts for the four Gaussians (*i* = 1–4) used in the fit. The peak height (*h*_i_) is given in arbitrary units. The uncertainties in the parameter values from the fits are given in parentheses.

sample	ω_1_	γ_1_	*h*_1_	ω_2_	γ_2_	*h*_2_	ω_3_	γ_3_	*h*_3_	ω_4_	γ_4_	*h*_4_

(A)	1039.49 (0.12)	15.20 (0.30)	4.94 (0.08)	1082.84 (3.91)	30.66 (7.14)	1.23 (0.17)	1092.46 (0.10)	1.73 (0.27)	2.00 (0.24)	1098.52 (5.05)	12.93 (20.31)	0.18 (0.39)
(B)	1040.94 (0.16)	18.76 (0.36)	9.92 (0.14)	1078.12 (1.12)	36.73 (3.74)	2.59 (0.11)	1092.48 (0.20)	2.64 (0.50)	2.45 (0.37)	1099.53 (0.48)	8.12 (1.51)	1.93 (0.27)
(C)	1040.69 (0.06)	15.06 (0.15)	32.19 (0.26)	1082.81 (1.29)	30.58 (2.11)	10.01 (0.40)	1092.46 (0.03)	1.73 (0.08)	21.62 (0.82)	1098.51 (0.48)	12.95 (1.88)	5.83 (1.07)

**Table 2:** Amplitude ratios *h*_TO_/*h*_TA_ and *h*_TO_/*h*_NP_ of the NW TA and NP lines, respectively, relative to the strongest line of the three NW lines (the NW TO mode) for each sample.

sample	*h*_TO_/*h*_TA_	*h*_TO_/*h*_NP_

(A)	4.02 (0.62)	27.44 (59.90)
(B)	3.83 (0.22)	5.14 (0.79)
(C)	3.22 (0.15)	5.52 (1.06)

Using the results obtained from the fits to the sample with the strongest PL (sample (C)), the NW TA and TO phonon energies are found to be 15.7 (1.8) and 57.8 (0.6) meV, respectively, which agree very well with the values expected for bulk Si_1−_*_x_*Ge*_x_* with *x* = 0.15 of 18 (1) and 58 meV, respectively [35]. The measured NW free-exciton NP energy of 1099 meV would indicate a Ge concentration of *x* = 0.14 (this concentration gives an X-point energy gap of 1099 meV in bulk SiGe) [35]. Thus the positions in energy of the NP peak and those of the accompanying phonon replicas independently confirm the alloy concentration as being *x* = 0.15 ± 0.01.

## Conclusion

The readily-observed PL seen from the SiGe NWs indicates they are clean (i.e., contain few growth defects and impurities) and are electrically isolated from the substrate. They are not strained to any significant extent and x for these samples is confirmed from the PL to be 0.15. These NWs with their well-controlled position, composition, and size and their efficient luminescence exhibit relevant features that are a significant improvement in quality over those produced by other vapor-solid-solid growth methods and that could be useful for applications in optoelectronic nanodevices. However, their mass production in current CMOS production lines would be problematic.
